# Molecular characterization, tissue expression and polymorphisms of buffalo *PPARGC1A* gene

**DOI:** 10.5194/aab-63-249-2020

**Published:** 2020-07-22

**Authors:** Lihua Qiu, Xinyang Fan, Yongyun Zhang, Xiaohong Teng, Yongwang Miao

**Affiliations:** 1 Faculty of Animal Science and Technology, Yunnan Agricultural University, Kunming 650201, Yunnan, China; 2 Teaching Demonstration Center of the Basic Experiments of Agricultural Majors, Yunnan Agricultural University, Kunming 650201, Yunnan, China

## Abstract

PPARGC1A exerts important functions in activating many nuclear receptors and transcription factors that are related to energy balance. Previous studies have shown that *PPARGC1A* gene is associated with lactation traits of dairy cattle. However, the functional role of the buffalo *PPARGC1A* gene is still unknown. In this work, the complete coding sequence (CDS) of buffalo *PPARGC1A* was isolated and characterized for swamp and river buffalo. The CDS length of *PPARGC1A* for both types of buffalo was the same, which was composed of 2394 nucleotides and encoded a peptide composed of 797 amino acid residues. This protein belonged to a hydrophilic protein and contained one RRM_PPARGC1A domain (AA 674–764) without a signal peptide or a transmembrane domain. The differential expressions of this gene in 10 buffalo tissues in lactation and non-lactation displayed that the *PPARGC1A* was highly expressed in the muscle, heart, liver, brain and kidney of both non-lactating and lactating periods, but its expression was significantly different in the muscle, heart, liver, small intestine, mammary gland, rumen, spleen and lung between the two periods. Eight single nucleotide polymorphisms (SNPs) were found in buffalo, in which the c.778C>T, c.1257G>A and c.1311G>A were shared by two types of buffalo with similar allele frequencies, while the c.419C>T, c.759A>G, c.920C>A, c.926G>A and c.1509A>T were only observed in river buffalo. The SNP419, SNP920 and SNP926 were non-synonymous, which led to the amino acid changes of p.Ser140Phe, p.Pro307His and p.Arg309Lys. Seven nucleotide differential sites were identified in the *PPARGC1A* gene between buffalo and other Bovidae species. Phylogenetic analysis indicated that buffaloes were independently clustered into one branch, but they were closely related to the species of the *Bos* genus. The results indicate that buffalo PPARGC1A is an inducible transcriptional coactivator involved in regulating carbohydrate and fat metabolism. It can exert a functional role in a variety of buffalo tissues and may participate in milk fat synthesis and development in the mammary gland.

## 1 Introduction

Peroxisome proliferation-activated receptor γ (PPARγ), as a main modulator of adipocyte differentiation, regulates the expression of genes related to fatty acid and glucose metabolism (Oberkofler et al., 2002). PPARγ coactivator-1α (PPARGC1A) interacts with PPARγ, permitting this protein to interact with various transcription factors, and then plays a role in multiple biological processes (Esterbauer et al., 1999; Knutti et al., 2000). The *PPARGC1A* gene was first identified in the brown fat cDNA library of mice in 1998 (Puigserver et al., 1998). Some researches have shown that PPARGC1A is responsible for activating thermogenesis and oxidative metabolism of brown fat and muscle (Spiegelman et al., 2000). Adenoviral-mediated expression of *PPARGC1A* strongly activates the whole process of key gluconeogenic enzymes in hepatocytes, indicating that PPARGC1A is a pivotal modulator of gluconeogenesis in the liver (Yoon et al., 2001). Cattle* PPARGC1A* gene is located on chromosome 6, containing 13 exons, with a total complete coding sequence (CDS) length of 2391 bp (Weikard et al., 2005). Some studies have shown that the *PPARGC1A* gene is closely related to milk protein (Pasandideh et al., 2015) and milk fat yield (Weikard et al., 2005; Chen et al., 2017) in cattle and goat. In addition, PPARGC1A is also a key regulator related to cattle intramuscular fat (Ramayo-Caldas et al., 2014).

Domestic buffalo can be divided into swamp buffalo and river buffalo. The former is mainly used for draft, with an annual milk production of 500–600 kg, while the latter is mainly used for milk production, with an annual milk production of about 2000 kg. The content of milk fat and protein in buffalo milk is significantly higher than that in the milk of dairy cattle (D'Ambrosio et al., 2008), which enables good processing characteristics for buffalo milk. Because the *PPARGC1A* gene has been proven to exert an important function in the expression regulation of genes related to fatty acid and glucose metabolism in some mammals, it can be used as a key candidate gene for lactation in dairy animals. In particular, the single nucleotide polymorphisms (SNPs) in this gene, which have a significant effect on milk production traits, can be used as a marker for assisted selection of buffalo lactation traits. But so far, there are few studies on the buffalo *PPARGC1A* gene. The purpose of this study is to isolate complete CDS of the buffalo *PPARGC1A* gene, to describe its molecular characteristics and multi-tissue differential expression in lactating and non-lactating stages, and to detect and characterize the SNPs in the CDS of this gene for two types of buffalo. This work will serve as a molecular basis to bringer further insight into the characteristics, functions and variation of the buffalo *PPARGC1A* gene.

## 2 Materials and methods

### 2.1 Animals and sampling

The heart, liver, spleen, lung, kidney, muscle, mammary tissue samples of adult healthy Binglangjiang buffaloes (river type, n=3) and Dehong Buffaloes (swamp type, n=3) were collected for gene isolation. After the buffalo had been slaughtered, each tissue sample was separated immediately, put into a freezing tube and stored in liquid nitrogen.

Eight Binglangjiang buffaloes (about four years old) – four in peak lactation (about 60 d postpartum) and four in dry-off period (about 60 d before parturition) – were selected for the collection of the samples for analysis of tissue differential expression. All buffalo sampled were managed in a similar fashion. After the buffalo were slaughtered, the tissue samples from the heart, liver, spleen, lung, kidney, mammary gland, small intestine, rumen, muscle and brain were immediately culled and stored in a refrigerator at -80 ${}^{\circ }$C until RNA extraction.

Furthermore, the fresh blood samples were collected from 108 Binglangjiang buffaloes and 81 Dehong buffaloes at a local breeding farm for population variation detection. The buffaloes used for sample collection were all adult healthy buffaloes without direct blood relationship.

All procedures for sample collection were performed in accordance with the Guide for Animal Care and Use of Experimental Animals approved by Yunnan Provincial Experimental Animal Management Committee under contract no. 2007-0069.

### 2.2 RNA extraction and cDNA synthesis

Total RNA was extracted from the buffalo tissues following the manufacturer's instructions for TRIzol reagent (Thermo Fisher Scientific, USA). The RNA was incubated with RNase-free DNase I (TaKaRa, China) to eliminate genomic DNA contamination. RNA quality of different tissues was assessed using agarose gel electrophoresis. Their concentrations were determined with the NANODROP LITE spectrophotometer (Thermo Fisher Scientific). The RNA (3 µg) was synthesized to cDNA through a First Strand cDNA Synthesis Kit (TaKaRa).

### 2.3 Cloning of the full-length CDS of the *PPARGC1A*

Base on the mRNA sequence of *Bos taurus* *PPARGC1A* (accession no. NM_177945), a pair of primers (Table 1) were designed to clone the whole CDS of the buffalo *PPARGC1A* gene by Primer Premier 5.0 (Lalitha, 2000). The 25 µL reaction system was as follows: 0.6 µM of each primer, 100 ng of cDNA template (mixed cDNA from each tissue) and 12.5 µL of 2xGoldStar MasterMix (Dye) (CWBIO, Beijing, China). The PCR program initially started with 95 ${}^{\circ }$C denaturation for 10 min, followed by 34 cycles of 95 ${}^{\circ }$C for 30 s, 57.5 ${}^{\circ }$C for 40 s, 72 ${}^{\circ }$C for 90 s, then 72 ${}^{\circ }$C extension for 5 min. The PCR products were detected by agarose gel electrophoresis. After gel extraction, the products were cloned into pMD-18T vector (TaKaRa) and sequenced bidirectionally using the Sanger method using ABI PRISM^®^ BigDye^®^ Terminator v3.1 Cycle Sequencing Kit (ABI, USA) on an ABI PRISM 3730 DNA sequencer according to the manufacturer's manual.

### 2.4 Physicochemical characteristics and structure analysis

The obtained sequence of buffalo *PPARGC1A* was checked, proofread and edited by the Lasergene software package (DNAstar Inc., USA). The open reading frame (ORF) was confirmed by Editseq (DNAstar Inc). Then, the homologous search was carried out to identify gene attributes by the BLAST program (https://blast.ncbi.nlm.nih.gov/Blast.cgi, last access: 20 March 2020) in the NCBI database. Physicochemical characteristics, hydropathy, signal peptide and transmembrane region were predicted by the ProtParam tool (https://web.expasy.org/protparam/, last access: 20 March 2020), ProtScale (https://web.expasy.org/protscale/, last access: 20 March 2020) SignalP-5.0 Server (http://www.cbs.dtu.dk/services/SignalP/, last access: 20 March 2020; Almagro-Armenteros et al., 2019) and TMHMM version 2.0 (http://www.cbs.dtu.dk/services/TMHMM/, last access: 20 March 2020), respectively. The conserved domain was determined through the Conserved Domain Architecture Retrieval Tool in BLAST. The subcellular localization was analyzed by ProtComp 9.0 (http://linux1.softberry.com/berry.phtml, last access: 16 March 2020). Secondary structures of deduced amino acid (AA) sequences were analyzed by SOPMA (http://npsa-pbil.ibcp.fr/, last access: 20 March 2020). Biological process and molecular function analysis was further conducted by InterProScan (http://www.ebi.ac.uk/interpro/search/sequence-search, last access: 21 March 2020).

### 2.5 RT-qPCR and tissue differential expression

To analyze tissue differential expression, a pair of primers were designed according to the obtained CDS of buffalo *PPARGC1A* in this work. The relative expression of *PPARGC1A* in 10 tissues during lactation and non-lactation were assayed by qPCR fluorescent technology using SYBR Premix Ex Taq (Takara) and performed on iQ5 Real Time PCR (Bio-Rad, USA) according to the manufacturers' instructions. The 20 µL reaction system included 2 µL cDNA, 10 µL SYBR Premix Ex Taq, 0.5 µL of 10 µM forward primer, 0.5 µL of 10 µM reverse primer, and 7 µL sterile water. The qPCR amplification was carried out firstly at 95 ${}^{\circ }$C for 30 s, then followed by 40 cycles of 95 ${}^{\circ }$C for 5 s, 60 ${}^{\circ }$C for 20 s and 72 ${}^{\circ }$C for 30 s. The β-actin (*ACTB*; accession no. NM_001290932) was used as an endogenous reference for normalization of *PPARGC1A* expression profiles (Table 1). The data of qPCR were analyzed through the 2${}^{-\Delta \Delta \mathrm{Ct}}$ method, where $\Delta \mathrm{Ct}={\mathrm{Ct}}_{\mathrm{PPARGC}1A}-{\mathrm{Ct}}_{\mathrm{ACTB}}$ and $\Delta \Delta \mathrm{Ct}=\Delta \mathrm{Ct}-\Delta {\mathrm{Ct}}_{\mathrm{median}}$. All the treatments were replicated three times. Significance of *PPARGC1A* mRNA level in multiple tissues between two periods was determined via Student's t test, which was established at a p<0.05.

**Table 1:** Primer information on gene isolation, qPCR and polymorphism identification.

	Primers (5${}^{\prime }$ to 3${}^{\prime }$)${}^{*}$	Product	Annealing	Usage
		length (bp)	temperature (${}^{\circ }$C)	
*PPARGC1A*	F: AACAGCTTGATTGGCGTCAT	2450	57.5	CDS isolation
	R: TATTCACCATGCCTCTGTCATCCTT			
*PPARGC1A*	F:CCACCGAGAATGAGGCTAGTCCTT	223	60	Differential expression
	R:TTGACAAATGCTCTTCGCTTTATTGCTCCA			
*ACTB*	F: TGGGCATGGAATCCTG	196	60	Differential expression
	R: GGCGCGATGATCTTGAT			
Exon 1	F: ACAGGTGCCTTCAGTTCA	254	56.1	SNP detection
	R: CCAAACCCAAGCCCTTCC			
Exon 2	F: TTTTCTCCCTGCCTCCTG	300	52.0	SNP detection
	R: CAAAGCAAGAACCCATTA			
Exon 3	F: TACTCATCTCCCAGTGTCA	300	53.7	SNP detection
	R: AGCCAGAGGCAACTCCAA			
Exon 4	F: CTCGCTTTCCCTCCTTCT	204	51.0	SNP detection
	R: AACCTCCTTGTGACTTCC			
Exon 5	F: TTCCCTTTCTTTATGCCT	278	53.1	SNP detection
	R: CCTCACCACCCTTACCAG			
Exon 6-7	F: CTGTTTCCAGTTTCCAAC	366	50.2	SNP detection
	R: ACACTCATCCATTCAAAA			
Exon 8	F: ATCTCAGGGAAGTGAGGAA	1158	54.1	SNP detection
	R: CACCAGGAACATGCTGTTGAG			
Exon 9	F: AGTCATGCTGATAAACTGGGTT	212	55.5	SNP detection
	R: GGATAAGAGGCACGGAGG			
Exon 10	F: AAAATGTAGTCCAAAACC	309	50.7	SNP detection
	R: TAATCTATGCCCATCACA			
Exon 11	F: TGGCATCAGTGTCTTTCC	263	51.2	SNP detection
	R: ATTCCCATCCTGGTAATC			
Exon 12	F: TGCTAATGCTGCCTCACT	309	48.4	SNP detection
	R: GGTAAAAGGTAGTAATGG			
Exon 13	F: AGGGTACATCTGACCTGG	169	51.1	SNP detection
	R: ATGCCTCTGTCATCCTTAGCC			

${}^{*}$ Primer direction (F: forward; R: reverse).

### 2.6 DNA isolation and polymorphism identification

Genomic DNA from the blood samples was isolated following a previous protocol (Sambrock and Russell, 2001). All primers used to amplify the exons were designed according to the genome sequence of buffalo *PPARGC1A* (accession no. NC_037551; Table 1). The mixture and protocol of PCR reaction were the same as that of clone, only by changing the annealing temperature and extension time. The PCR products were bidirectionally sequenced using the corresponding PCR primers.

The mutation sites were confirmed and outputted using Seqman (DNAstar Inc.) and Mega 6 (Tamura et al., 2013). Allele and genotype frequency and Hardy–Weinberg equilibrium test were carried out using PopGen32 software (Yeh and Boyle, 1997). The function influence of non-synonymous substitutions was presumed by the program PANTHER (http://www.pantherdb.org/, last access: 21 March 2020; Mi et al., 2017). The haplotypes were inferred by PHASE (Stephens et al., 2001). The optimal maximum likelihood model was determined by model selection tests, and then the phylogenetic tree was established based on the Hasegawa-Kishino-Yano model with a bootstrap test of 10 000 replicates.

## 3 Results

### 3.1 Cloning and identification of buffalo *PPARGC1A*

**Figure 1:**
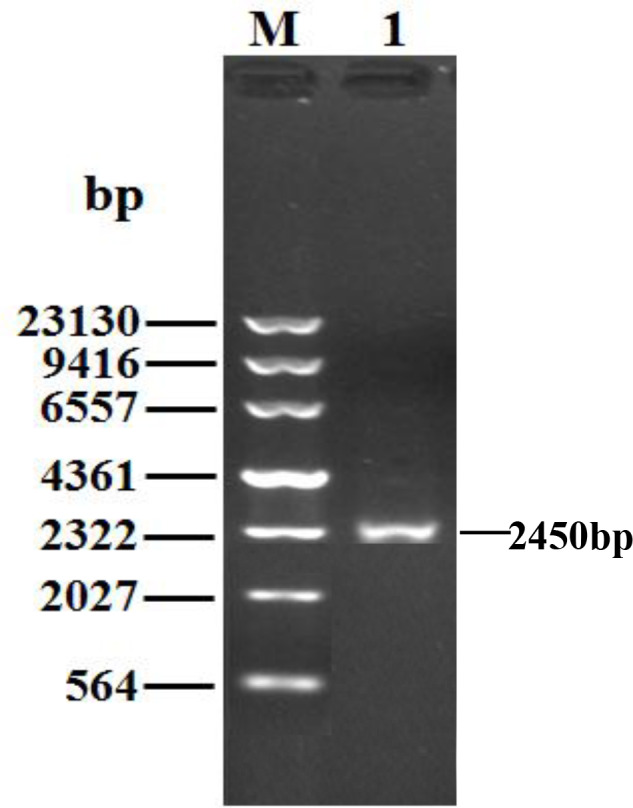
PCR results for the buffalo *PPARGC1A* gene. M, λDNA marker; 1, PCR product for the buffalo *PPARGC1A* gene.

**Figure 2:**
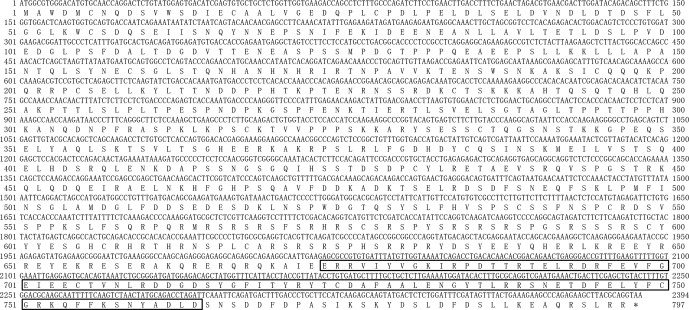
Nucleotide sequence of buffalo *PPARGC1A* and its deduced amino acid sequence. The predicted protein sequence is shown immediately above the nucleotide sequence. The RRM_PPARGC1A domain (AA 674–764) is boxed. The stop codon is indicated by an asterisk (${}^{*}$).

Being consistent with expectations, the PCR products of 2450 bp were obtained (Fig. 1). Sequence prediction showed that the cDNA sequence contained an ORF of 2394 bp. The homology search was performed by the BLAST program in the NCBI database, and the results displayed that the identity between the ORF sequence and the CDS sequences of the *PPARGC1A* gene in cattle (NM_177945), yak (XM_005897078), bison (XM_010839915), sheep (XM_004009738) and goat (NM_001285631) was 99.12 %, 99.21 %, 98.29 %, 99.00 % and 98.83 %, respectively. Sequence analysis displayed that the nucleotide sequence of the *PPARGC1A* of river buffalo was consistent with that of swamp buffalo. The overall base composition of buffalo *PPARGC1A* CDS was composed of 29.62 % A, 22.68 % G, 21.85 % T and 25.86 % C. Buffalo *PPARGC1A* was deduced to encode a protein consisting of 797 amino acids (AAs) (Fig. 2).

### 3.2 Characteristics and structures of the PPARGC1A protein

**Figure 3:**
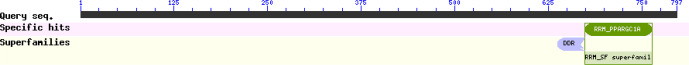
Putative conserved functional domain of buffalo PPARGC1A.

The comparison of physicochemical characteristics of PPARGC1A between buffalo and cattle (accession numbers AC_000163) is shown in Table 2. The physicochemical characteristics of buffalo PPARGC1A were similar to those of cattle. The molecular weight of buffalo PPARGC1A was about 90.49 kDa, and its theoretical isoelectric point was 6.06. This protein belonged to an unstable protein with an instability index (II) of 74.87. Its grand average of hydropathicity was -1.086, illustrating that buffalo PPARGC1A is a hydrophilic protein. Secondary structure analysis showed that the PPARGC1A consists of 31.37 % α-helix (250 AA), 11.67 % extended strand (93 AA), 4.27 % β turn (34 AA) and 52.70 % random coils (420 AA) (Fig. S1 in the Supplement). Furthermore, the buffalo PPARGC1A contained one conserved RRM_PPARGC1A (AA 674–764) functional domain (Fig. 3) without a signal peptide or a transmembrane region. Cytoplasm/nuclear localization analysis suggested that buffalo PPARGC1A was distributed in nucleus (35.4 %), plasma membrane (21.3 %), Golgi (17.9 %) and mitochondria (10.3 %).

**Table 2:** Physicochemical characteristics of PPARGC1A for buffalo and cattle.

Basic physical and chemical properties	Buffalo	Cattle
Formula	C${}_{3865}$H${}_{6072}$N${}_{1158}$O${}_{1302}$S${}_{28}$	C${}_{3857}$H${}_{6056}$N${}_{1152}$O${}_{1302}$S${}_{28}$
Number of amino acids	797	796
Molecular weight	90.49 kDa	90.30 kDa
Isoelectric point (pI)	6.06	5.92
Strongly acidic amino acid (D, E)	122	123
Strongly basic amino acid (K, R)	111	110
Polar amino acid (N, C, Q, S, T, Y)	281	281
Hydrophobic amino acid (A, I, L, F, W, V)	172	172
Instability index (II)	74.87	75.01
Grand average of hydropathicity (GRAVY)	-1.086	-1.082
Aliphatic index	53.14	53.08

### 3.3 Biological process and molecular function

Predictions showed that buffalo PPARGC1A was involved in the biological process of mitochondrial organization (GO:0007005) and brown fat cell differentiation (GO:0050873). Its molecular functions are mainly nucleic acid binding (GO:0003676), transcription factor binding (GO:0008134), signaling receptor binding (GO:0005102) and transcription coregulator activity (GO:0003712).

### 3.4 Tissue differential expression of the *PPARGC1A*

**Figure 4:**
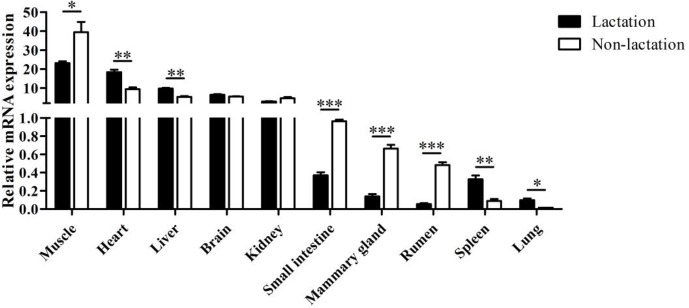
Tissue differential expression of buffalo *PPARGC1A *in 10 tissues during lactation and non-lactation. The values are presented as means ± SEM; ${}^{*}$ P<0.05, ${}^{**}$ P<0.01, ${}^{***}$ P<0.001.

The tissue differential expression of the *PPARGC1A* gene was assayed via qPCR in 10 tissues of lactating and non-lactating river buffalo (Fig. 4). The results indicated that the *PPARGC1A *gene was expressed in almost all tissues during these two periods, especially in the muscle, heart, liver, brain and kidney. However, the expression of this gene in the brain and kidney did not change significantly between lactation and non-lactation (P>0.05). The expression of the *PPARGC1A* gene in the heart, liver, spleen and lung during lactation was significantly higher than that during non-lactation (P<0.05), but its expression in the muscle, mammary gland, small intestine and rumen was on the contrary (Table S1 in the Supplement, Fig. 4).

### 3.5 Population variation analysis

Five synonymous SNPs were found in the samples of this study, namely c.759A>G, c.778C>T, c.1257G>A, c.1311G>A and c.1509A>T (Table 3). The c.759A>G and c.1509A>T were found only in river buffalo, and the others were shared by two types of buffalo. The information on each SNP is shown in Table 3. The c.778C and c.1257G were the alleles with high frequency in two types of buffalo. It is worth noting that SNP1311 in swamp buffalo were all heterozygous. The test of Hardy–Weinberg equilibrium showed that SNP778 in both types of buffalo and SNP1311 in swamp buffalo were in dis-equilibrium (P<0.05).

**Table 3:** Genetic information on the SNPs found in two types of buffalo.

Population	SNP	Genotype	Frequency	Allele	Frequency	P value${}^{*}$
River buffalo	c.759A>G	AA	0.500	A	0.7083	0.9012
		AG	0.417	G	0.2917	
		GG	0.083			
	c.778C>T	CC	0.750	C	0.7500	0.0002
		CT	0.000	A	0.2500	
		TT	0.250			
	c.1257G>A	GG	0.667	C	0.8333	0.5556
		GA	0.333	T	0.1667	
		AA	0.000			
	c.1311G>A	GG	0.083	G	0.4167	0.2542
		GA	0.667	C	0.5833	
		AA	0.250			
	c.1509A>T	AA	0.917	C	0.9583	1.0000
		AT	0.083	G	0.0417	
		TT	0.000			
Swamp buffalo	c.759A>G	AA	1.000	A	1.0000	–
		AG	0.000	G	0.0000	
		GG	0.000			
	c.778C>T	CC	0.778	C	0.7778	0.0007
		CT	0.000	A	0.2222	
		TT	0.222			
	c.1257G>A	GG	0.667	C	0.8333	0.6326
		GA	0.333	T	0.1667	
		AA	0.000			
	c.1311G>A	GG	0.000	G	0.5000	0.0047
		GA	1.000	C	0.5000	
		AA	0.000			
	c.1509A>T	AA	1.000	C	1.0000	–
		AT	0.000	G	0.0000	
		TT	0.000			

${}^{*}$ P value of Hardy–Weinberg equilibrium test.

**Figure 5:**
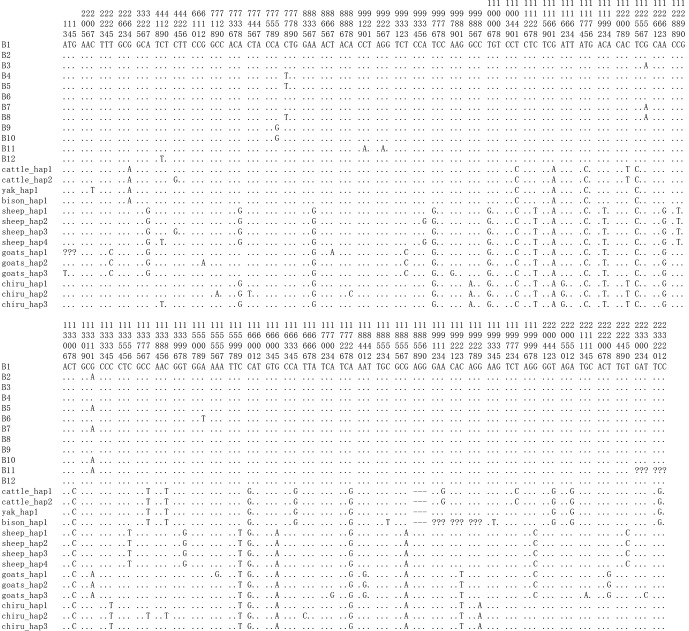
Nucleotide differences of the haplotype sequences among some species of Bovidae. Number represents the position of coding region. Dots (.) denote identity with the B1. Nucleotide substitutions are denoted by different letters. Missing information is denoted by a question mark (?). Horizontal line (–) represents the deletion in the sequences. These definitions apply to the following figure as well.

**Figure 6:**
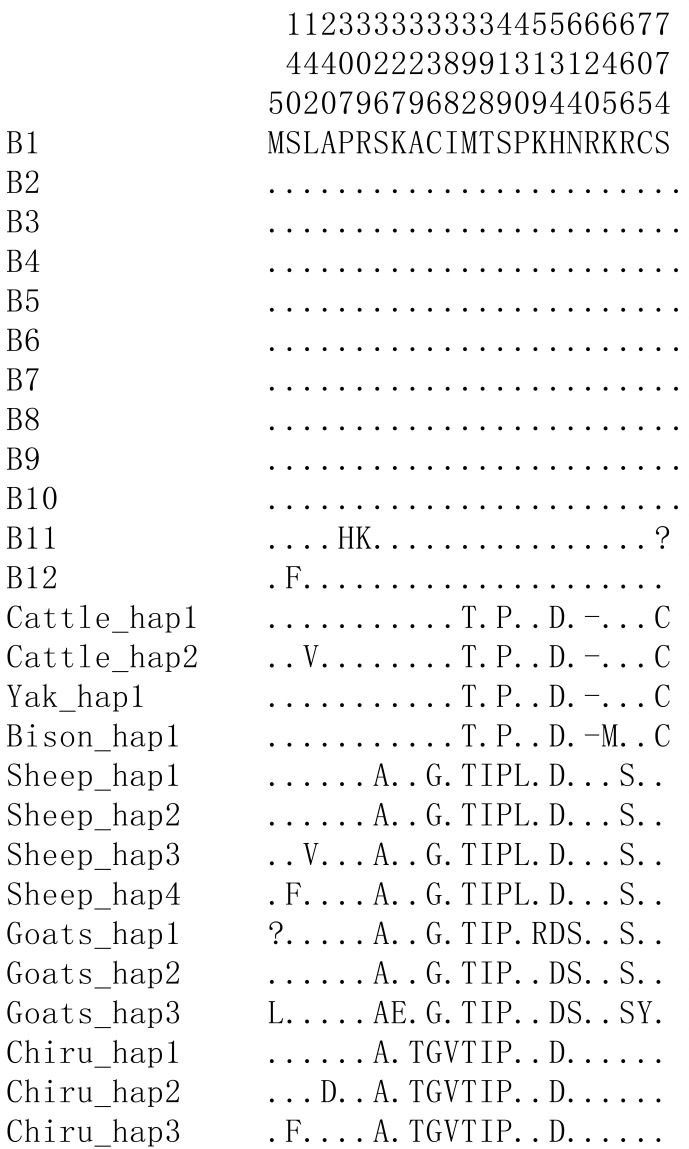
Differences of amino acid sequences corresponding to the haplotypes of *PPARGC1A* in the species of Bovidae.

By analyzing *PPARGC1A* gene sequences of buffalo obtained in this work and the published data of this gene in the NCBI database, we discovered that the number of SNP in buffalo *PPARGC1A* increased to eight. Among them, the c.419C>T, c.920C>A and c.926G>A were from published data in the NCBI database, which were only observed in river buffalo. They are all non-synonymous, resulting in amino acid changes of p.Ser140Phe, p.Pro307His and p.Arg309Lys in the PPARGC1A (Figs. 5 and 6). The prediction showed that the substitutions of p.Ser140Phe and p.Pro307His may affect the function of buffalo PPARGC1A (Table 4).

**Table 4:** Functional effect of non-synonymous substitution on buffalo PPARGC1A.

SNP	Substitution	Preservation	Message
		time	
c.419C>T	p. Ser140Phe	456	Probably damaging
c.920C>A	p. Pro307His	456	Probably damaging
c.926G>A	p. Arg309Lys	361	Possibly damaging

### 3.6 Haplotype sequence differences and phylogenetic relationship

Based on the polymorphisms of the *PPARGC1A* gene, a total of 12 haplotypes (B1–B12) were defined in two types of buffalo (Fig. 5). Among them, 10 (B1–B10) (accession numbers MN788077–MN788086) were obtained from the data of this work (Table 5), and the other two were from published data (accession numbers HQ236498 and NW_005785900). Among these haplotypes, B1–B5 were shared by two types of buffalo, and the rest were only found in river buffalo.

**Table 5:** Haplotype information on the buffalo *PPARGC1A* gene.

	Haplotype	Actual frequency	Expected frequency
B1	ACGGA	0.143	0.196
B2	ACGAA	0.310	0.267
B3	ACAGA	0.095	0.054
B4	ATGGA	0.071	0.095
B5	ATGAA	0.119	0.095
B6	ACGGT	0.024	0.011
B7	ACAAA	0.024	0.055
B8	ATAGA	0.048	0.024
B9	GCGGA	0.071	0.062
B10	GCGAA	0.095	0.094

In order to explore the sequence differences of the *PPARGC1A* gene between buffalo and other animals in Bovidae, all the haplotype sequences of buffalo in this work were compared with the published homologous sequences of other species of Bovidae. The accession numbers in the NCBI database of the representative haplotypes for each species are XM_010806009, AC_000163, XM_005897078, NW_011494708, NW_014639015, XM_012131592, NC_019463, NW_011942373, HM600810, NC_030813, EU304457, XM_005980241, NM_001286596 and NW_005815902. The sequence differences of nucleotide and its corresponding amino acid among all the species are shown in Figs. 5 and 6, respectively. There were seven nucleotide differences located at c.1041, c.1161, c.1175, c.1255, c.1308, c.1600 and c.1728 of this gene between buffalo and other species of Bovidae, including three nucleotide differences (c.1175, c.1255 and c.1600) which led to the amino acid differences of the PPARGC1A (the corresponding amino acids in buffalo PPARGC1A were p.392Met, p.419Ser and p.534His, respectively) (Fig. 6). It is noteworthy that the *PPARGC1A *gene of buffalo and the *Ovis* genus was three nucleotides longer (from c.1858 to c.1860) than that of *Bos* (with an Arg insertion at p.620 in buffalo and *Ovis*).

**Figure 7:**
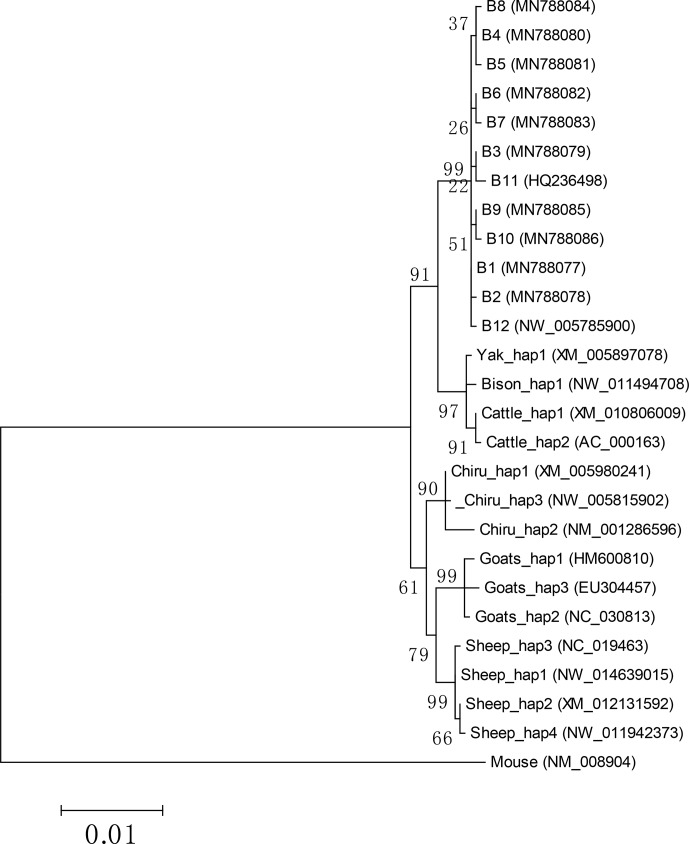
Phylogenetic tree constructed by using maximum likelihood method (Hasegawa-Kishino-Yano model). Bootstrap confidences which are adjacent to nodes are based on 10 000 replicates.

Based on the haplotype sequences of buffalo, cattle, yak, bison, sheep, goat and chiru, a phylogenetic tree was established with a homologous sequence of mouse as an outgroup (Fig. 7). The phylogenetic analysis showed that buffalo, *Bos* and *Ovis* gathered on their own independent clades with high supports. The genetic relationship between buffalo and the species of *Bos* is closer than that of the species in *Ovis*.

## 4 Discussion

In this work, the whole CDS of the *PPARGC1A* was cloned from two types of buffalo. The PPARGC1A for both river and swamp buffalo was all composed of 797 amino acid residues, and its basic physicochemical properties were similar to those of cattle. The prediction of subcellular localization showed that the buffalo PPARGC1A was distributed not only in the nucleus and plasma membrane, but also in the mitochondria and Golgi, indicating that buffalo PPARGC1A may exert a biological function in the nucleus, plasma membrane, mitochondria and Golgi. Previous studies have shown that PPARGC1A contains a RRM_PPARGC1A domain and is an inducible transcriptional coactivator that can interact with a variety of transcription factors, which are related to various biological processes, including adaptive thermogenesis, glucose/fatty acid metabolism, skeletal muscle fiber type switching and cardiac development (Spiegelman et al., 2000; Yoon et al., 2001; Mortensen et al., 2006). It has been confirmed that PPARGC1A can interact with steroid receptors and activate them (Knutti et al., 2000). It can also coordinate the expression of genes participated in fatty acid metabolism (Dominy et al., 2010) and is closely related to milk fat synthesis (Weikard et al., 2005; Chen et al., 2017). In this study, it is predicted that buffalo PPARGC1A also contains a RRM_PPARGC1A domain, and its molecular functions are mainly involved in nucleic acid binding, signal receptor binding, transcription factor binding and transcriptional coregulator activity. The sequence consistency, physicochemical properties, and structure of buffalo PPARGC1A were similar to those of cattle. Based on the above results, it is speculated that buffalo PPARGC1A is also associated with the regulation of carbohydrate and lipid metabolism, and it exerts a function in adipose tissue, skeletal muscle, heart, liver and mammary gland. These results also indicate that buffalo PPARGC1A may have similar functions to other mammals, especially the Bovidae species.

Previous studies have shown that the mouse *PPARGC1A* gene is highly expressed in brown fat, heart, kidney and brain, but it is lower in the liver and the lowest in white adipose tissue (Puigserver et al., 1998). And the human *PPARGC1A* is highly expressed in the heart, skeletal muscle and kidney, but its expression in the liver, brain, pancreas and perirenal adipose tissue is low (Esterbauer et al., 1999). In this study, the *PPARGC1A* gene was highly expressed in the muscle, heart, liver, brain and kidney of both lactating and non-lactating buffaloes, indicating that this gene exerts a key role in these buffalo tissues under various physiological conditions. The expression levels of buffalo *PPARGC1A* in the muscle, heart, liver, small intestine, mammary gland, rumen, spleen and lung between the two periods were significantly different, indicating that the expression of this gene in these tissues was regulated by physiological state. In the present study, the expression of the *PPARGC1A* gene was detected in the mammary gland of lactating buffalo, and it is speculated that this gene also plays a role in milk fat synthesis of buffalo. It is noteworthy that buffalo *PPARGC1A* was expressed in the mammary gland with a higher expression level during the dry-off period than that during peak lactation. This may be due to the fact that the *PPARGC1A* gene also participates in the degradation and remodeling of the mammary gland tissue in the non-lactating buffaloes. It has been reported that there are quantitative trait loci influencing milk traits in dairy cows on BTA 6, and the *PPARGC1A* gene is located in the middle of BTA 6 (Khatib et al., 2007). Therefore, this gene is also qualified as a key functional candidate gene influencing milk production traits (Weikard et al., 2005). In addition, by constructing the network of genes involved in milk fat synthesis, it is revealed that bovine PPARGC1A plays a pivotal regulatory function in the network of milk fat synthesis in the mammary gland (Bionaz and Loor, 2008).

So far, multiple polymorphic sites have been found in the cattle *PPARGC1A* gene. In dairy cows, there were significant association between c.1790+514G>A, c.1892+19T>C and c.1892+19G>A of *PPAGC1A* gene and milk fat yield (Weikard et al., 2005; Schennink et al., 2009). However, there are few reports about the SNPs of the *PPARGC1A* gene in buffalo. In this work, a total of eight SNPs were found in buffaloes, of which three were non-synonymous. And it was predicted that two non-synonymous substitutions (c.419C>T and c.920C>A) led to changes in amino acids of p.Ser140Phe and p.Pro307His, which seriously affected the function of buffalo PPARGC1A. The Ser is a polar AA, while the Phe is a hydrophobic AA. The Pro is a non-polar AA, while the His is a basic AA. These substitutions belong to amino acid substitutions with different physicochemical properties. In addition, the 140Ser is a potential O-glycosylation site and this substitution may lead to the loss of an O-glycosylation site of buffalo PPARGC1A. These may cause changes in the structure or function of buffalo PPARGC1A, while SNPs, which cause synonymous changes, are thought to act sometimes by altering translation efficiency and thus can influence traits (Zhou et al., 2018). Whether the SNPs identified in this study, especially the non-synonymous SNPs, have any effect on the function of buffalo PPARGC1A and the lactation traits of buffalo needs to be verified by further expanding the sample size.

The alignment indicated that the CDS length of buffalo *PPARGC1A* was 3 bp longer than that of *Bos* but the same as that of *Ovis*. In addition, there were three amino acid differential sites in the PPARGC1A between buffalo and other species of Bovidae. It is speculated that these differences in PPARGC1A may lead to functional differences between buffalo and other species of Bovidae. The phylogenetic tree showed that buffalo had a closer genetic relationship with the species of *Bos*. This indicates that there may be little difference in the function of PPARGC1A between buffalo and the species of *Bos*. In addition, there were 13 and 9 differential nucleotides in the *PPARGC1A* gene between buffalo and *Bos* and between buffalo and *Ovis*, respectively, which can be used as molecular markers to distinguish buffalo from other species of Bovidae.

## 5 Conclusions

The length of *PPARGC1A* CDS for both types of buffalo was the same, which encoded a peptide composed of 797 amino acid residues with the same physicochemical properties and molecular functions. Buffalo PPARGC1A contains one RRM_PPARGC1A domain without a signal peptide or a transmembrane domain and is an inducible transcriptional coactivator related to the regulation of carbohydrate and lipid metabolism. It can function in a variety of tissues and performs a critical function in the milk fat synthesis of the mammary gland. Eight SNPs were found in two types of buffalo. However, only the p.Ser140Phe and p.Pro307His caused by c.419C>T and c.920C>A may affect the function of buffalo PPARGC1A. The mechanism of buffalo PPARGC1A on milk traits is still unknown and needs to be further analyzed. This work will lay a preliminary foundation for further understanding the structure and function of the buffalo PPARGC1A.

## Supplement

10.5194/aab-63-249-2020-supplement The supplement related to this article is available online at: https://doi.org/10.5194/aab-63-249-2020-supplement.

## Data Availability

The original data used in this study are available from the corresponding author upon request.
