# Supplementary material for: Molecular characterization, tissue expression and polymorphisms of buffalo PPARGC1A gene
Source: Arch Anim Breed. 2020 Jul 22;63(2):249–59. doi: 10.5194/aab-63-249-2020 (PMC7405651; doi:10.5194/aab-63-249-2020)
Supplement: The supplement related to this article is available online at: https://doi.org/10.5194/aab-63-249-2020-supplement. [file aab-63-249-supplement.pdf]

Supplement of Arch. Anim. Breed., 63, 249–259, 2020
https://doi.org/10.5194/aab-63-249-2020-supplement
© Author(s) 2020. This work is distributed under
the Creative Commons Attribution 4.0 License.

Open Access
Archives Animal Breeding

Supplement of

Molecular characterization, tissue expression and polymorphisms of buffalo *PPARGC1A* gene

Lihua Qiu et al.

Correspondence to: Yongwang Miao (yongwangmiao1@126.com)

The copyright of individual parts of the supplement might differ from the CC BY 4.0 License.

Table S1. Data information for tissue differential expression of buffalo *PPARGC1A* gene

Tissues	Relative expression level (mean ± standard error)*		*P* values
	lactation	non-lactation	
Muscle	23.1816 ± 0.9303	39.3687 ± 5.4650	0.0432
Heart	18.3880 ± 1.3240	9.4338 ± 0.8944	0.0050
Liver	9.7518 ± 0.2973	5.3388 ± 0.4343	0.0011
Brain	6.4906 ± 0.3496	5.5206 ± 0.1164	0.0580
Kidney	2.8722 ± 0.2056	4.6320 ± 0.5773	0.1038
Small intestine	0.3704 ± 0.0348	0.9644 ± 0.0181	0.0001
Mammary gland	0.1404 ± 0.0239	0.6643 ± 0.0404	0.0004
Rumen	0.0558 ± 0.0097	0.4843 ± 0.0293	0.0002
Spleen	0.3268 ± 0.0410	0.0899 ± 0.0204	0.0066
Lung	0.0989 ± 0.0166	0.0150 ± 0.0004	0.0297

*The values are the average values for each tissue in four lactating or four non-lactating buffaloes.

```
         10        20        30        40        50        60        70
          |         |         |         |         |         |         |
MAWDMCNQDSVWSDIECAALVGEDQPLCPDLPELDLSELDVNDLDTDSFLGGLKWCSDQSEIISNQYNNE
eeeecccttchhhhhhhhhhhcccccccccccccccccccccccchhhhhhhcccccchhhhhhhhcccc
PSNIFEKIDEENEANLLAVLTETLDSLPVDEDGLPSFDALTDGDVTTENEASPSSMPDGTPPPQEAEEPS
cchhhhhcchhhhhhhhhhhhhhhhtccccctttccchhecctteceeccccccccccccccccccccchh
LLKKLLLAPANTQLSYNECSGLSTQNHANHNHRIRTNPAVVKTENSWSNKAKSICQQQKPQRRPCSELLK
hhhhhhhccccceeechhtccccccccccccccccccceeeeehcchhhhhhhhhhtccccccchhhhhh
YLTTNDDPPHTKPTENRNSSRDKCTSKKKAHTQSQTQHLQAKPTTLSLPLTPESPNDPKGSPFENKTIER
heeccccccccccccccccccccccccccccccchhhhhtcccceeecccccccccccccccccccchhh
TLSVELSGTAGLTPPTTPPHKANQDNPFRASPKLKPSCKTVVPPPSKKARYSESSCTQGSNSTKKGPEQS
eeeeeettcccccccccccccccccccccccccccttcceecccccccceeccccccccccccccccccch
ELYAQLSKTSVLTSGHEERKAKRPSLRLFGDHDYCQSINSKMEILVSTSQELHDSRQLENKDAPSSNGSG
hhhhhhccceeeeecccccccteeeecccccchhhhhhhhhhheeehhhhhhhhhhcccccccccccccc
QIHSSTDSDPCYLRETAEVSRQVSPGSTRKQLQDQEIRAELNKHFGHPSQAVFDDKADKTSELRDSDFSN
ceeecccccchhhhhhhhhhhcccttcccchhhhhhhhhhhhhtccccchhhhhhtcccchhhhhhttcch
EQFSKLPMFINSGLAMDGLFDDSEDESDKLNSPWDGTQSYSLFHVSPSCSSFNSPCRDSVSPPKSLFSQR
hhhhhcceeecthhhhhhhhcccccchhhhcccccccceeeeeeecccccccccccccccccccceehcc
PQRMRSRSRSFSRHRSCSRSPYSRSRSRSPGSRSSSRSCYYYESGHCRHRTHRNSPLCARSRSRSPHSRR
cchhhhtcccccccccccccccccccccccccccccccceeeeettccccccccccccccccccccccccc
PRYDSYEEYQHERLKREEYRREYEKRESERAKQRERQRQKAIEERRVIYVGKIRPDTTRTELRDRFEVFG
cccchhhhhhhhhhhhhhhhhhhhhhhhhhhhhhhhhhhhhhhhteeeeeecccccccchhhhhhhhhhhh
EIEECTVNLRDDGDSYGFITYRYTCDAFAALENGYTLRRSNETDFELYFCGRKQFFKSNYADLDSNSDDF
hhhhheeeeccttcceeeeeeecchhhhhhhhttceecccccccheeeeetcceeecttteeecccccccc
DPASIKSKYDSLDFDSLLKEAQRSLRR
cchhhcccchhhhhhhhhhhhhhhhhh
```

Figure S1. Predicted secondary structure of buffalo PPARGC1A. Alpha helices, extended strands, beta turns, and random coils are indicated with the h, e, t, and c, respectively.
